# One-Piece Titanium Implants: Retrospective Case Series

**DOI:** 10.1155/2021/6688355

**Published:** 2021-04-08

**Authors:** Gunar Wagner, Dieter Hartung

**Affiliations:** ^1^Department of Restorative Dentistry and Periodontology, University Medical Center Leipzig, 04103 Leipzig, Germany; ^2^Dental Practice, 37213 Witzenhausen, Germany

## Abstract

**Purpose:**

One-piece titanium implants are not routinely used for reconstruction after tooth loss. Several limitations seemed to be apparent although the concept provides a straightforward approach for different clinical situations. A clinical documentation of five prosthetic restorations with one-piece titanium implants serving as a relevant treatment option in dental surgery is pursued. We demonstrate the feasibility and benefits of one-piece titanium implants for fixed dental prosthesis. Detailed descriptions of the technical features and the surgical approach by means of clinical cases are given. The prosthetic workflow when working with one-piece titanium implants is depicted in detail as well as examples for implant-supported tooth replacement in the posterior region and the esthetic zone. Conditions of applications regarding different timing of implant placement using the system and its limitations are discussed.

**Results:**

Clinical cases with a follow-up period of up to 10 years are presented to prove the long-term success of one-piece titanium implants in terms of bone and soft-tissue stability respecting the biological criteria for periodontal health.

**Conclusions:**

One-piece titanium implants represent a reliable treatment method for single-tooth replacements. Clinical success with long-time bone stability around the implantation site can be achieved. Taken into account the requirements for periodontal tissue stability, uneventful healing without extensive tissue loss is demonstrated by means of clinical cases presenting patients with periodontitis.

## 1. Introduction

Although dental implant therapy can be considered a standard procedure, various biological and technical aspects must be considered to avoid incidence and progression of peri-implant mucositis and peri-implantitis [1]. Clinical success of dental titanium implants to preserve marginal bone and soft-tissue levels is still one of the most commonly studied subjects in modern dentistry leading to the emergence of various technical and surgical developments [2–4]. The majority of dental implants used in the clinical field remain two-piece implants. The implant-abutment interface of two-piece implants is usually positioned at the epicrestal or subcrestal level. Frequently bone loss of 1.5-2 mm vertically and 1.5 mm horizontally can be observed around dental implants. This is widely accepted if later bone loss within one year after loading does not exceed 0.2 mm annually [5–7]. Nonetheless, peri-implantitis progresses in a nonlinear accelerating pattern and the majority of cases occur within 3 years of function [8]. Several factors are being discussed to be of significant importance to maintain peri-implant health. Hard- and soft-tissue deficiencies can lead to complications and impaired implant survival [9]. The clinical circumstances in patients with periodontitis are challenging and should be taken into account during implant therapy. A common threat for recession formation around dental implants is posed by repeated changes of the implant-abutment interface [10, 11]. To stabilize the soft-tissue thickness around dental implants, platform-switching concepts or the one abutment-one time protocol was developed to eliminate the potential for disturbances to the peri-implant mucosal barrier [3, 12]. One-piece titanium implants were originally introduced to the market with a sturdy design for minimally invasive and conventional prosthetic methods enabling restoration of single-tooth defects. Due to high primary stability, it was proposed that early loading using immediate implant placement could therefore be performed [13, 14]. The absence of a microgap and abutment complications remains favourable in the oral cavity [15]. The macroconfiguration design of one-piece titanium implants facilitates high insertion torque in situations with varying bone density. This could be of importance considering the vulnerable implant-abutment connection in two-piece implants leading to technical failures. This concept was not further extended due to lower success rates and evident bone loss after one year within a specific titanium implant system [16, 17]. Within the limitations of the current case series, different clinical situations are used to illustrate recent developments and benefits introducing the one-piece titanium implant system FairOne™ (FairImplant GmbH, Bönningstedt, Germany) to achieve periodontal stability and long-term success.

## 2. One-Piece Implant System

**Figure 1:**
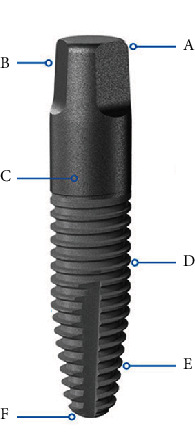
Features of the one-piece conical titanium implant FairOne™: (a) preparable head: 3°, (b) three prosthetic flanks for immediate impressions or digital scanning processes, height: 3.5 mm; (c) transition zone with continuous rough surface (1.5-2.5 *μ*m), height: 4 mm; (d) surface with resorbable CaP-coating (BONIT®), roughness: 4 *μ*m, conical shape for high primary stability; (e) self-tapping threads and congruent drills for iBIC; (f) round apex designed to protect anatomical structures.

The one-piece titanium implant FairOne™ (FairImplant GmbH, Bönningstedt, Germany) was introduced to the market in 2006. The macro geometry and the surface design are constructed with an emphasis on periodontal soft-tissue integration and peri-implant stability to achieve best clinical results. The conical-shaped implant made of titanium medical grade four is composed of self-tapping threads and congruent drills. Three prosthetic flanks at the coronal part ensure precise immediate impression or performance of digital scanning processes. Taken into account the complex interplay between scalloped bone topography and different gingival profiles, this one-piece implant is designed with a continuous rough surface promoting long-term clinical stability [18, 19]. Lack of an implant-abutment connection with formation of a microgap is beneficial for one-piece implants since this could promote adherence of inflammatory cells accompanied with a significant peri-implant bone loss [20]. The moderately rough surface at the prosthetic head promotes an adequate transition from soft- to hard-tissue connection which is aimed at combining undisturbed osseointegration and periodontal healing [21]. Osteoconduction on both etched and commercially pure titanium surfaces could be significantly increased with modified nanoscale deposits of calcium phosphate crystals on the implant surface [22, 23]. Therefore, implant threads are additionally coated with a resorbable calcium phosphate layer for improved osseointegration (BONIT®, DOT GmbH, Rostock, Germany). The conical shape of the implant body was designed to achieve high primary stability [24]. The FairOne™ is designed with a round apex to work as a bone condenser and to protect surrounding anatomical structures. Maxillary sinus lift procedures could be facilitated by preservation of the fragile mucosa (Figure 1).

**Figure 2:**
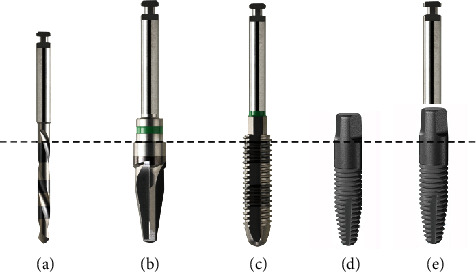
(a) Predrilling instrument ø 2.0 mm; (b) shaping-drill with conical design for bone harvesting during preparation, variable diameter and length; (c) thread cutter (optional); (d) one-piece implant (FairOne™) with self-tapping thread; (e) overlapping view of shaping-drill with corresponding tapered design of the FairOne™ (B+D).

A drilling-protocol with depth and diameter conformed instruments was specially developed for this system. During osteotomy, a shaping-drill with conical design corresponding to the tapered design of the implant is able to avoid unnecessary damage of osseous structures during implant bed preparation. Additionally, given the design of the drill, it is easy to harvest autologous bone chips with high quality at the site of osteotomy. When necessary, a thread cutter allows for low insertion-torque during preparation but in the large majority of cases, the self-tapping threads on the implant surface allow for ideal primary stability (Figure 2).

When placing implants, a continuous stability during the healing phase is one of the key factors for success. Usually after three to four weeks, primary stability is minimized due to increased osteoclastic activity around implants. New bone formation leading to secondary stability has not yet occurred, and the overall stability receives a significant drop [25]. The bone metabolic activity could also be significantly influenced depending on the magnitude and period of loading [26]. The one-piece implant and the concerted drilling protocol are aimed at achieving an optimized initial bone-implant contact (iBIC) to facilitate immediate implant placement.

## 3. Clinical Case 1: Surgical Procedure Placing One-Piece Implants

**Figure 3:**
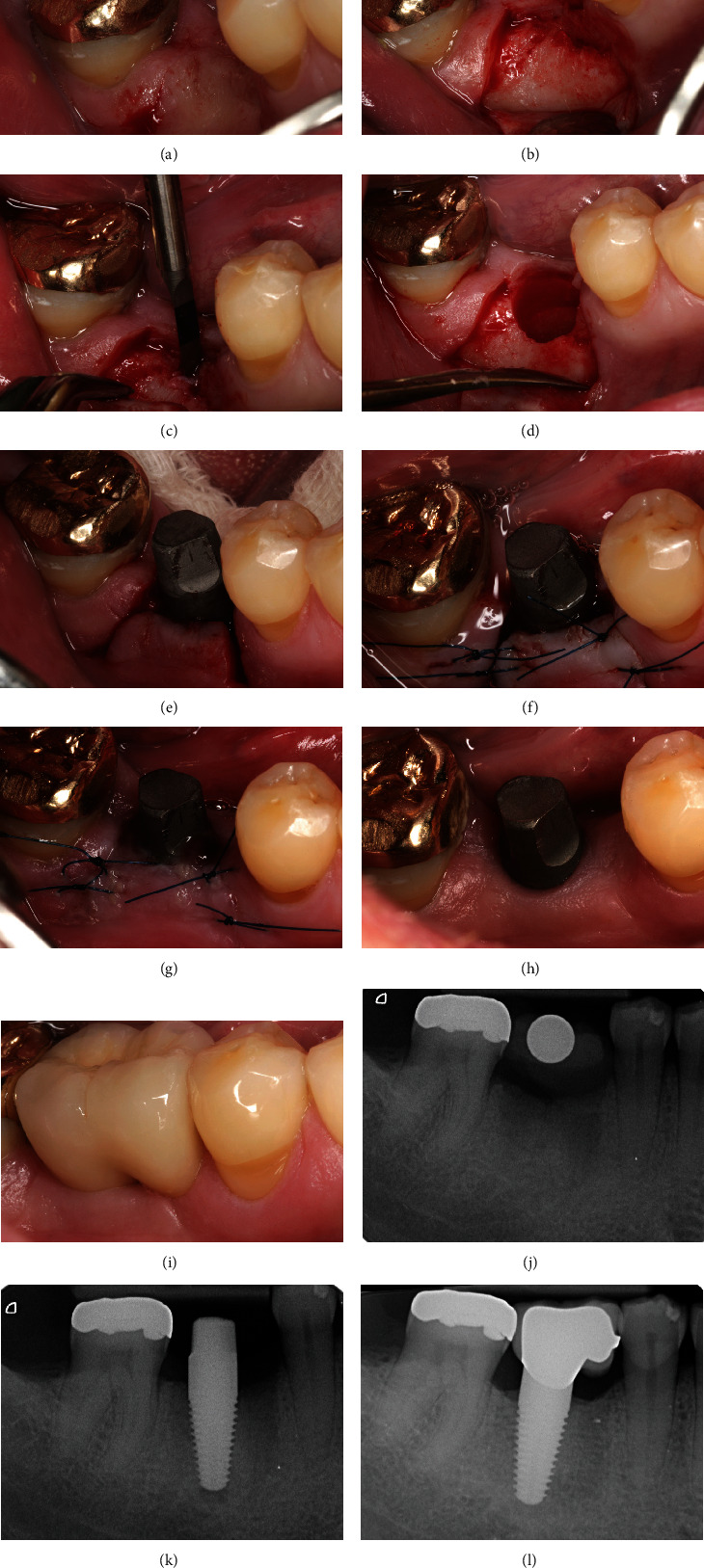
Implantation of one-piece implant (clinical procedure): (a) clinical situation presurgically; (b) elevation of full-thickness flap with preservation of peripheral periodontium at adjacent teeth; (c) implant bed preparation, predrilling instrument; (d) processed implantation site using drilling protocol; (e) placement of one-piece implant with spatial orientation aligned to the natural dentition; (f) apical repositioned and sutured flap; (g) clinical situation one week after surgery; (h) clinical situation 12 weeks after surgery; (i) single crown prosthetic restoration with mesial pontic design; (j) presurgical radiographic control, calibration ball for planning of implantation; (k) postsurgical radiograph taken directly after implant placement; (l) radiographic control nine years after treatment.

In the following case, a 60-year-old, healthy male patient received restorative therapy with a one-piece implant after tooth loss. The patient has undergone periodontal treatment in the past. Keratinized gingiva around dental implants is important for long-term success of the rehabilitation. If the tissue thickness is 2.0 mm or less, crestal bone loss up to 1.45 mm may occur [27]. The amount of KG at the desired site for implantation was sufficient, and surgery could be performed without soft-tissue grafting. Bone sounding was performed to assess the defect morphology presurgically and provide sufficient information for the design of the flap (Figure 3(a)). The surgical procedures were performed using a microsurgical approach. Surgical access was obtained by preparation of a trapezoidal full-thickness flap. Particular attention was paid in the preservation of the adjacent soft tissue. The oral portion of the interdental periodontal complex was left undetached. The flap was gently elevated to the buccal site before implantation (Figure 3(b)). After implant-bed preparation using the drilling-protocol, a one-piece implant (FairOne™; diameter: 5.0 mm and length: 10 mm) was placed (Figures 3(c) and 3(d)). The insertion torque of 50 Ncm was applied. Special attention should be paid since the buccal area of the bone crest is usually positioned more apically than the mesial and distal flanks of the implant site. The flap was apically repositioned and sutured in a tension-free condition (Seralon®, D1S 15, 5-0, Serag-Wiessner, Naila, Germany) providing a sufficient buccal preservation of at least 4 mm of KG at the healing site (Figures 3(e) and 3(f)). After one week of nonsubmerged healing, the surgical site showed proper conditions showing no signs of recession formation (Figure 3(g)). Sutures were removed postsurgically, and the patient was asked to abstain from mechanical oral hygiene in the area for two weeks. A chlorhexidine mouth rinse CHX 0.2%, (Dynexidin® forte, Kreussler & Co. GmbH, Wiesbaden, Germany) was used for chemical biofilm control. After 12 weeks of implantation, the surgical site showed perfect conditions for single-tooth replacement with a thick KG around the implant (Figure 3(h)). The prosthetic treatment could be finalized using a metal-supported ceramic. The crown was designed with a mesial cantilever achieving a pontic design in order to allow for a proper interdental cleaning (Figure 3(i)). The postinterventional maintenance protocol included professional biofilm debridement at 1-month intervals up to 3 months postoperatively, followed by a recall visit at 6 months postoperatively. Motivation for personal plaque control was reinforced at each recall visit. The radiographic evaluation showed no signs of bone resorption and a biological stable situation even after nine years of implant treatment (Figures 3(j)–3(l)). The patient had not experienced complications during the procedure and recovered without complaints.

## 4. Clinical Case 2: Demonstrating the Prosthetic Workflow Using FairOne™

**Figure 4:**
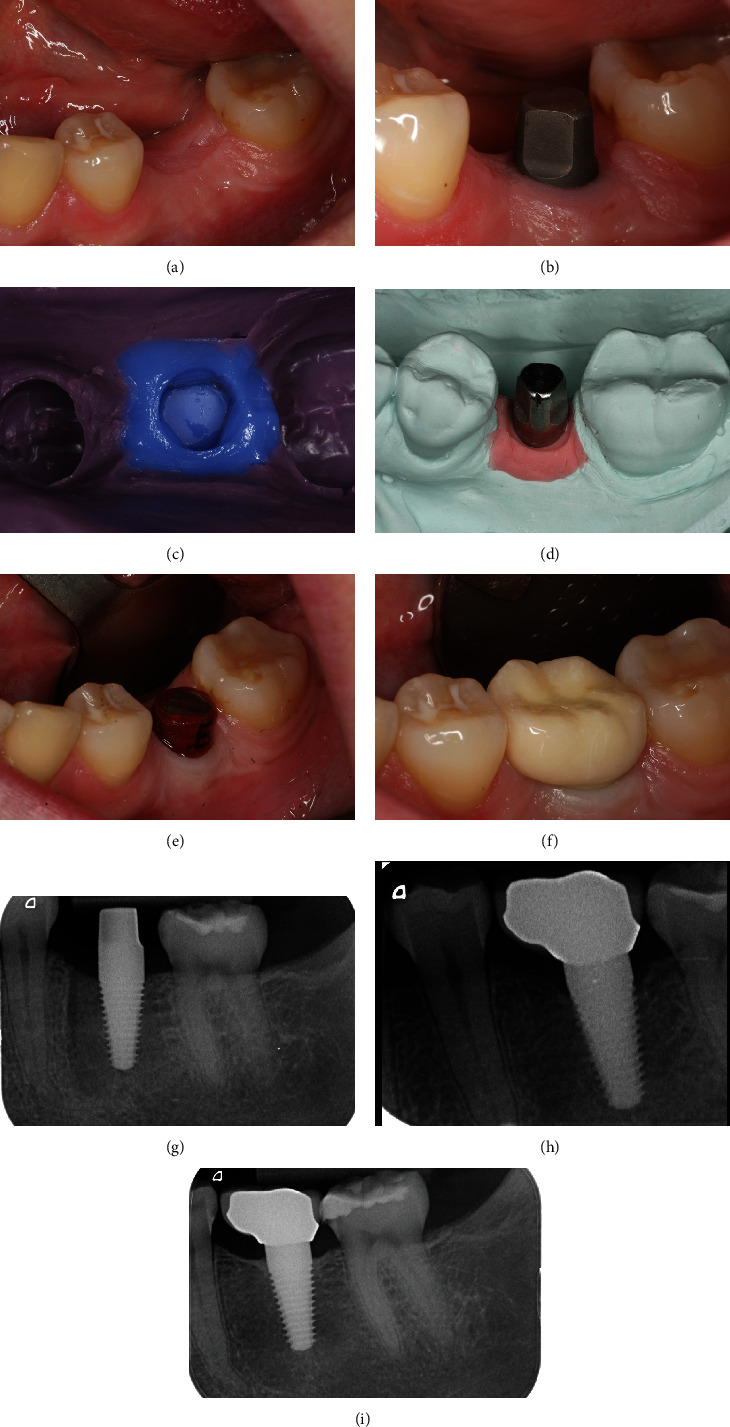
Prosthetic workflow for the one-piece implant FairOne™: (a) clinical situation presurgically presenting sufficient keratinized gingiva around implant site; (b) clinical situation after healing period of 12 weeks; (c) impression with double-mix technique; (d) laboratory processing, master cast with implant analog, and gingival mask; (e) clinical preparation using a transfer coping for occlusal adaption; (f) clinical situation with cemented implant crown (mesial pontic design); (g) postoperative radiographic control after delayed implantation; (h) radiographic evaluation four months after prosthetic restoration; (i) radiographic control nine years after implant treatment.

For single crown restorations, the following case is aimed at illustrating the prosthetic procedure. A 23-year-old, healthy female patient diagnosed with periodontitis suffered from apical periodontitis after root canal treatment on tooth 36. Due to a hopeless prognosis, a decision was made to extract the tooth and to use delayed implantation with a one-piece implant. The clinical situation after atraumatic extraction showed no signs of inflammation and sufficient quantity of soft tissue (Figure 4(a)). Assessment of the correct position and dimensions for the planned reconstruction was realized using a radiograph with calibration ball. After implantation with a one-piece implant (FairOne™; diameter: 5.0 mm and length: 10 mm), it was ensured that no interference in the static or dynamic occlusion is occurring during healing. Minimal adjustments can be performed by means of the preparable head. In order to achieve accurate transmission for the prosthetic restoration, the geometric design should be preserved. This provides the possibility for direct impressions or digital scanning with high-detail reproduction and precision. In the posterior region, no provisional restoration was necessary during the healing phase, since there is no additional surgery for uncovering of the implant and no abutment dis-/reconnection shaping of the emergence profile occurred early during the initial healing phase on the continuous rough surface (Figure 4(b)). Additionally, the baseline of the bone crest in the mesial and distal direction is documented (Figure 4(g)). The impression could be performed using a double-mix technique with a polyether (Impregum™, 3M, Landsberg am Lech, Germany) (Figure 4(c)). Within the laboratory process, a master cast in conjunction with an implant analog and gingival mask was used adjusting the prosthetic restoration and ensuring that the emergence profile of the crown is optimally contoured (Figure 4(d)). In cases were the clinical situation causes interference with the crown design, a transfer coping joined with pattern resin is fabricated. This could enable the surgeon to realize defined adjustments for the prosthetic head prior to the crown fitting and cementation process (Figure 4(e)). CAD/CAM processing is possible with the use of a digital implant model analog (CAM-Analog, FairImplant GmbH, Bönningstedt, Germany). The final restoration was placed using a metal-supported ceramic crown (Figure 4(f)). Provisional cementation for up to four weeks securing the final fitting of the contact situation in the approximal area and adjustment of the occlusal situation as well as the color of the crown was conducted with eugenol-free temporary cement (TempBondNE™, Kerr GmbH, Bioggio, Switzerland). The permanent cementation was usually conducted using a dual-curing dimethacrylate composite with a custom analog for precementation to significantly reduce excess cement (PANAVIA F™, Kuraray Noritake, Tokyo, Japan). Implant placement should ideally by controlled by periapical radiographs that were taken by the use of a long-cone paralleling technique. After 10 weeks of implant treatment, a slight loss in bone height adjacent to the implant in the mesial and distal direction could be observed but no excess of cement is evident (Figure 4(h)). The patient reported no complications during the procedure and the healing period. Nine years after implant placement, remineralisation and formation of new bone at the peri-implant interface can be observed presenting stable biological conditions (Figure 4(i)).

## 5. Clinical Case 3: Soft-Tissue Augmentation around One-Piece Implants

**Figure 5:**
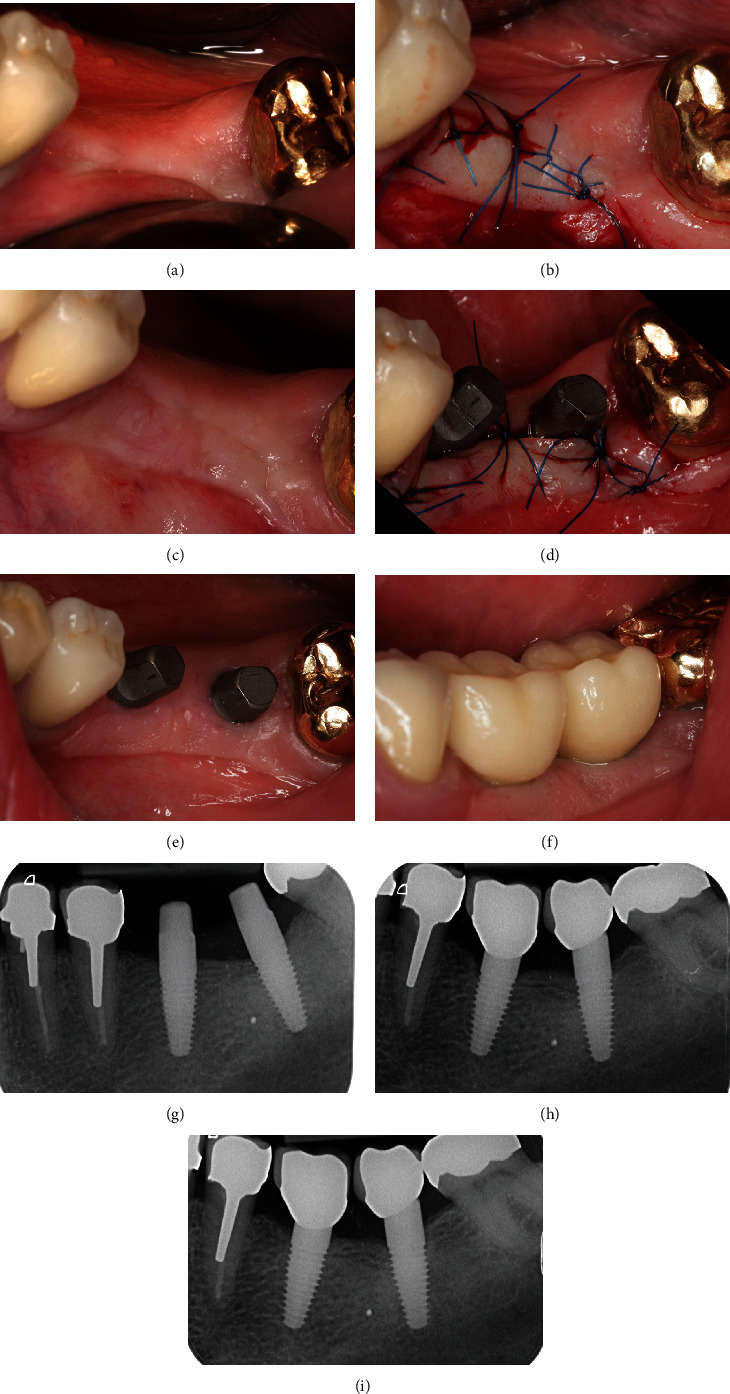
Soft-tissue augmentation in the region of the planned implantation: (a) clinical situation presurgically presenting small alveolar crest and reduced width of keratinized gingiva around implant site; (b) augmentation of free gingival graft at the buccal aspect; (c) clinical situation six weeks after healing; (d) postoperative clinical situation after implantation of two one-piece implants, flap closure; (e) clinical situation after 12 weeks of transgingival healing; (f) prosthetic restoration six months after implantation, cemented single crowns; (g) radiographic control after implantation; (h) postsurgical radiograph taken directly after cementation of single crowns; (i) radiographic control nine years after implant treatment.

Single-tooth replacements of interdental spaces or edentulous areas can be achieved with one-piece implants. In the following case, a 50-year-old, healthy male patient who experienced periodontal treatment in the past, demonstrating a thin alveolar crest, was treated with one-piece titanium implants at the left side of the mandible. The keratinized tissue around the targeted implant site was reduced (Figure 5(a)). After augmentation of a free gingival graft (FGG) harvested from the palate, the buccal aspect of the recipient site could be notably expanded to assure thick gingival conditions with keratinized tissue (Figures 5(b) and 5(c)). After uneventful healing of the graft, implant placement with adequate alignment of two implants could be performed (FairOne™; diameter: 4.2 mm and length: 10 mm). An insertion torque of 30 Ncm was applied. A tension-free suturing was conducted (Seralon®, D1S 15, 5-0, Serag-Wiessner, Naila, Germany) providing a sufficient buccal preservation of at least 4 mm of KG at the healing site (Figure 5(d)). After implantation, postsurgical radiographs were taken with a proper parallel technique to monitor the bone crest and correct positioning of the implants (Figure 5(g)). After a healing period of thirteen weeks, successful periodontal integration could be documented (Figure 5(e)). Splinting of the two implants was performed using a flowable composite (Tetric Evo Ceram®, Ivoclar Vivadent, Schaan, Lichtenstein) to support osseointegration. Implants were restored with metal-supported ceramic crowns to allow for full recovery of the masticatory function within the jaw segment (Figure 5(f)). No patient reported complaints could be noticed during the whole procedure. To control for excess cement and proper positioning of the crown, a radiographic control was performed (Figure 5(h)). Nine years after implant placement, a stable osseous situation could be documented and the clinical situation does not indicate the presence of peri-implantitis (Figure 5(i)).

## 6. Clinical Case 4: Delayed Implantation in the Esthetic Area

**Figure 6:**
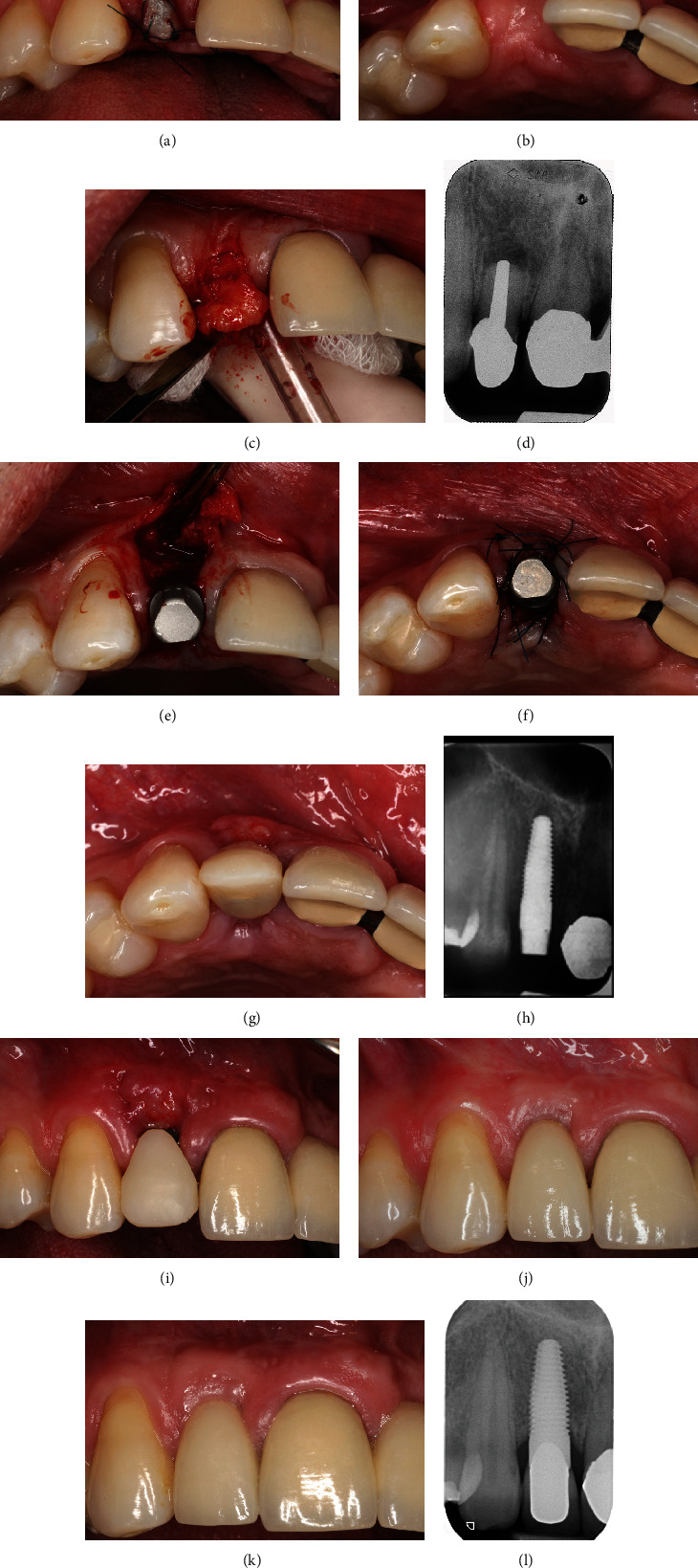
Delayed implantation of one-piece implant in esthetic zone: (a) clinical situation after extraction and socket preservation with soft-tissue augmentation; (b) clinical situation presurgically presenting small horizontal dimension of alveolar ridge; (c) reflection of connective tissue pedicle using roll technique with implant placement; (d) initial radiographic situation with hopeless tooth; (e) clinical situation after implant placement; (f) suturing and fixation of connective tissue at the buccal site; (g) healing after one week and suture removal, immediate provisional restoration (occlusal view); (h) radiographic control after implantation; (i) healing period one week after implantation with provisional restoration (buccal view); (j) prosthetic restoration with single crown four months after healing; (k) clinical situation 10 years after intervention; (l) radiographic control 10 years after implant treatment.

The following case illustrates the performance of one-piece titanium implants for complete functional and esthetic rehabilitation in the esthetic zone. A 49-year-old male patient suffering from glaucoma and hypertension was seeking help at the dental practice with complaints in the upper jaw. After radiographic control, the maxillary right lateral incisor was diagnosed with chronic apical periodontitis following root canal treatment. The tooth had a hopeless prognosis but the surrounding bone was assessed to be appropriate for implantation (Figure 6(d)). Subsequent to minimal invasive extraction, socket preservation with soft-tissue augmentation by means of a free gingival graft was conducted (Figure 6(a)). Healing occurred uneventful, and soft-tissue dimensions could be preserved (Figure 6(b)). The implantation was performed three months after extraction. To support the alveolar ridge during implantation, a connective tissue pedicle using the roll technique was performed [28]. The connective tissue was prepared from the palatinal area and rotated to the buccal site of the implantation site (Figure 6(c)). After implantation of the one-piece implant (FairOne™; diameter: 5.0 mm and length: 10 mm) (Figure 6(e)), the flap was repositioned and tension-free suturing was secured (Figure 6(f)). A temporary provisional composite crown for esthetic demands was delivered directly after implantation (Figure 6(g)). After implantation, postsurgical radiographs were taken with a proper parallel technique to monitor the bone crest and correct positioning of the implants (Figure 6(h)). One week after uneventful healing, sutures could be removed with interdental papilla remained well preserved (Figure 6(i)). The patient reported only slight pain directly postoperative. The composite crown was replaced with a metal-supported ceramic crown, and after four months of healing, stable soft-tissue conditions without recession formation could be documented (Figure 6(j)). The clinical and radiological situation remained stable even 10 years after intervention with a good prognosis (Figures 6(k) and 6(l)).

## 7. Clinical Case 5: Immediate Implantation in the Esthetic Area

**Figure 7:**
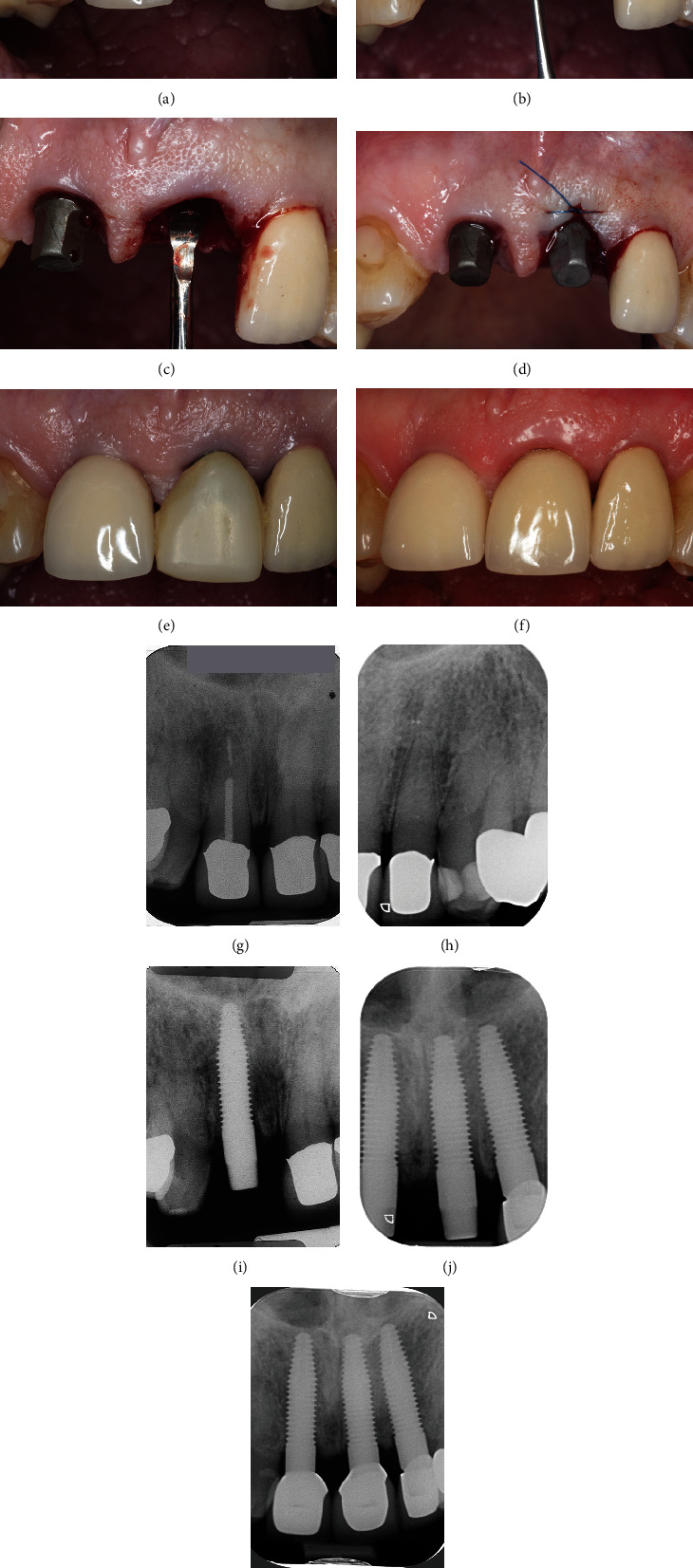
Immediate restoration of one-piece implant in esthetic zone: (a) clinical situation after fracture of left central incisor, (b) atraumatic extraction of remaining tooth, (c) complete extraction and cleaning of alveolar ridge, (d) clinical situation after immediate implant placement, (e) provisional restoration, reattachment of implant crown for right central incisor, (f) prosthetic restoration with single crown five months after healing, (g) initial radiographic situation with hopeless right central incisor, (h) radiographic situation before implantation of left lateral incisor, (i) radiographic control after implantation of first right incisor, (j) radiographic control after immediate implantation of left central incisor, and (k) radiographic situation nine months after immediate implant placement for left central incisor.

Immediate placement using one-piece titanium implants could be part of routine clinical practice. After fracture of the maxillary left central incisor, a 54-year-old, healthy patient who received periodontal treatment in the past was administered to implant therapy using FairOne™. The clinical situation does not support any symptoms of severe infection or traumatic damages of the surrounding soft tissue (Figure 7(a)). Preoperative radiographs provide information about the osseous environment prior to implant therapy. These served as a baseline image to monitor the height of the bone to implant contact for further diagnosis and prevention of peri-implantitis (Figures 7(g) and 7(h)). The patient decided for immediate implant therapy after discussing treatment options. During atraumatic minimal invasive extraction, debonding of the neighboring implant crown took place presenting healthy soft-tissue conditions (Figure 7(b)). After complete extraction, a precise cleaning and rinsing of the extraction socket was conducted (Figure 7(c)). Immediate placement using a one-piece titanium implant (FairOne™; diameter: 4.2 mm and length: 13 mm) was performed, and complete wound closure could be carried out establishing primary stability (Figure 7(d)). A temporary provisional composite crown for esthetic demands was delivered, and rebonding of the adjacent implant crown was conducted. Appropriate design of the provisional restoration was considered to preserve soft-tissue stability around the implants during healing period (Figure 7(e)). Following healing, a temporary metal-supported ceramic crown could be provided five months after implant therapy (Figure 7(f)). The patient was satisfied and had no complaints during the whole procedure. The radiographic control showed stable osseous conditions for the one-piece implant of the maxillary first central incisor (Figure 7(i)). Eight years after the first implantation, a second one-piece implant had to be inserted for the left lateral incisor. A postsurgical radiograph taken directly after immediate implantation of the left central incisor did not show further bone loss evident in the area of interest. The preceding interventions using FairOne™ at adjacent teeth are clinically and radiologically inconspicuous (Figure 7(j)). The radiographic control of nine months after immediate implantation revealed bone stability for all one-piece titanium implants (Figure 7(k)).

## 8. Discussion

Critical evaluations revealed lower success rates and more bone resorption for one-piece implants with direct loading [16]. It must be considered that the clinical success for restorations using one piece-implants is determined by the implant design, insertion depth, and loading protocols. Marginal bone loss around two-piece implants of more than 0.44 mm/year could be an indication of peri-implant bone loss progression and should be avoided [29]. The fact that transgingival healing of one-piece implants represents a risk factor for microbial infection by direct contact via pathogens from the oral cavity at the mucogingival border is a common argument. In contrast, no differences between nonsubmerged and submerged healing in terms of significant soft-tissue loss could be revealed [15]. Furthermore, experimental studies concluded that the biological width (BW) dimensions for one-piece implants are favourable compared to two-piece implants and were more similar to the natural dentition [30]. Taken into account the criteria for periodontal stability, we could demonstrate successful healing of one-piece implants in the posterior region without loss of the BW resulting in long-term success. Disadvantages discussed leading to impairments of peri-implant health are the inevitable cementation process of one-piece implants without the possibility of screw-retained restorations. In this context, one has to take into account that two-piece implants with prefabricated abutments and a plain platform connection in the crestal area could not compensate the natural course of the peri-implant soft-tissue outline or the scalloped alveolar leading to situations where the cementation gap is positioned much deeper subgingivally than necessary. Consequently, the crown margin does not remain accessible for cleansing and oral hygiene resulting in increased probability for cementation-associated peri-implantitis [31]. Although recent evidence found no significant influence on the positioning of the implant regarding the alveolar crest [32], bacterial colonization of the implant-abutment junction could be facilitated with a subcrestal positioning of the implant [33]. Correct positioning of the crown margin at the level of the mucosal margin could easily be achieved with one-piece implants. Through the continuous rough surface of the FairOne™, possible resorption processes occurring in the early phase of healing could be prevented, as the alignment of the implant-abutment interface with the bone crest is not required. It is important to note that excessive cleansing after cementation of the implant-crown and postsurgical radiographic documentation is recommended not only to control for excess cement but also to document the baseline conditions of the surrounding bone [34]. Systematic analysis revealed that soft-tissue thickness around implants is a decisive factor for early remodeling. In contrast to natural teeth, implants have to be supported with approximately 4 mm of vertical soft tissue as the minimum required height for biological stability [24]. Lack of attached gingiva around dental implants could otherwise lead to increased inflammation and the occurrence of mucositis [35]. Hence, there is a higher risk of gingival recession around implants, and marginal bone loss is more likely to occur [36, 37]. This could additionally lead to patient discomfort especially during brushing and hygiene [38]. In order to prevent soft-tissue dehiscence's caused by the lack of limited peri-implant soft-tissue thickness and the absence of adequate keratinized mucosa, a consequent periodontal management using mucogingival surgery to boost the biotype should be a prerequisite for dental implants [39, 40]. A stable soft-tissue seal as protection barrier from a contaminated intraoral environment is an important factor stabilizing the BW around dental implants [41]. Soft-tissue augmentation remains recommended if necessary when using one-piece implant as demonstrated. Additionally, a significant influence of the presence/absence of a microgap between the implant and the abutment is a decisive factor [42]. X-ray microtomographic imaging on different types of implant-abutment joints reveals bacterial penetration that could be responsible for peri-implant pathologies [43, 44]. Considering these findings, one-piece implants without the formation of a microgap could possibly reduce the bacterial load at critical sites. Repeated abutment disconnections and reconnections considerably increased marginal bone loss and buccal recession in two-piece implants [11, 45]. Data from meta-analyses proofed that less bone resorption and soft-tissue shifts occur by preventing repeated abutment changes for platform switched implants [46]. Further investigations demonstrated a superior method to compensate such peri-implant tissue changes by using one-time abutments [47]. These findings support the idea of undisturbed healing and periodontal integration provided by one-piece dental implants. When placing immediate implants, the goal is to achieve the most beneficial initial bone-implant contact (iBIC) enabling a transition from primary mechanical stability, provided by the design of the implant body, to biologic stability due to new bone formation. Therefore, the application of an ideal insertion torque is of particular interest when placing implants. High insertion torque (≥40 Ncm) was discussed to cause strong compression and distortion in the peri-implant bone leading to necrosis of the osteocytes due to disturbed microcirculation. In contrast, clinical and radiographical studies proofed that high insertion torque does not impair crestal bone levels [48]. Although small cracks at the implant sites could be revealed histologically, no bone resorption was detected supporting the possibility of increased remodeling processes that occur to repair microcracked bone [49]. In this context, the application of one-piece implants with high insertion torque of at least 32 Ncm could be recommended and is supported by the design of the FairOne™. This is of particular interest when performing immediate implant placement securing primary stability even in challenging situations. However, a recent systematic review demonstrates that loading protocols are not likely to influence the clinical outcomes, including implant survival and peri-implant stability regarding implant therapy for single tooth [50]. One-piece implants could meet the requirements for esthetic success providing a scalloped gingival line similar to natural teeth. Nevertheless, in situations where primary stability could not be achieved, one-piece implants have limitations. In such cases, a submerged healing using a two-piece implant would be the treatment of choice. Spatial positioning with profound backward planning is decisive for the prosthetic treatment as the implant system is limited for modifications compared to an individual abutment system using two-piece implants. Systematic analysis for one-piece implants revealed long-term survival rates similar to two-piece designs. Nonetheless, technical and biological complications are still being observed frequently for both systems [51, 52]. The design and the surgical guidelines of the FairOne™ seek to overcome those challenges.

## 9. Conclusions

One-piece implants proved to be a useful tool for implant-supported tooth replacement in the posterior region as well as in the esthetic zone with benefits regarding the implant design and the surgical workflow. We could demonstrate long-term results of up to 10 years proving that preservation of the biological width including the level of the bone crest to achieve periodontal stability can be realized. Patients who received periodontal treatment in the past are usually compromised due to extensive attachment loss or higher susceptibility for peri-implantitis. Surgical treatment using one-piece titanium implants did not reveal increased tissue loss or frequency of peri-implant diseases in patients with periodontitis. Further clinical studies should evaluate the present findings in comparison with two-piece implant systems using profound clinical data and adequate observation periods.

## Data Availability

The authors confirm that the data supporting the findings of this study are available within the article.
